# Early Adoption of Digital Devices and Change in Loneliness Over 10 Years Among Late Middle–Aged and Older Adults: Longitudinal Cohort Study

**DOI:** 10.2196/95955

**Published:** 2026-09-22

**Authors:** Anna K Dahl Aslan, Martin Gellerstedt, Chandra A Reynolds, Deborah Finkel

**Affiliations:** 1 School of Health Sciences University of Skövde Skövde, Västra Götaland Sweden; 2 Institute for Behavioral Genetics and and Department of Psychology & Neuroscience University of Colorado Boulder Boulder, CO United States; 3 Institute of Gerontology Jönköping University Jönköping, Jönköping Sweden; 4 Center for Economic and Social Research University of California, Los Angeles Los Angeles, CA United States

**Keywords:** loneliness, computer use, computer access, cell phone use, cell phone access, digital health, older adults, old age, digitalization, epidemiology

## Abstract

**Background:**

Loneliness is recognized as a global health threat. Older adults are vulnerable to loneliness due to life changes common in old age. While individual risk factors for loneliness in old age are well-documented, contextual factors are scarcely explored, such as digitalization. Rapid digitalization underscores the need to explore the long-term association between the use of digital devices and loneliness.

**Objective:**

This study aimed to explore whether and how the use of digital devices is associated with changes in loneliness in a population-based sample of middle-aged and older adults in a context where digital technology and digital environments were introduced and rapidly evolving.

**Methods:**

Data were obtained from the longitudinal cohort study, the Swedish Adoption/Twin Study of Aging (SATSA; N=771; mean age 69.64 years, SD 11.00). Daily digital use and loneliness were assessed across 5 waves between 2004 and 2014. Age, sex, education, living situation, self-rated health, and the personality traits openness and extraversion were assessed at baseline. Growth mixture modeling was used to identify latent trajectories of loneliness, and multinomial logistic regression was used to predict class membership based on baseline daily digital use and covariates.

**Results:**

Three latent loneliness classes were identified: class 1 (10.5%; high intercept and significant increases in loneliness), class 2 (33.2%; intermediate stable loneliness), and class 3 (56.3%; low stable loneliness). Daily digital use at baseline is significantly associated with lower odds of belonging to the high-increasing loneliness group (class 1) compared to the low-stable group (class 3; odds ratio 0.27, 95% CI 0.08-0.90; *P*=.03). Differences between classes were not explained by the personality traits.

**Conclusions:**

Daily use of digital devices is associated with lower and more stable levels of loneliness over time, compared to not using digital devices daily, which is associated with higher and increasing levels of loneliness. These findings, based on data from early adopters of digital technology, lend further support to the idea that digital use is associated with lower levels of loneliness and less increase in loneliness. Hence, these findings suggest that use of digital devices may represent a noninvasive approach to addressing loneliness in older adults.

## Introduction

Loneliness is “a subjective unpleasant feeling of lack of or loss of companionship that happens when there is a mismatch between the quantity and quality of the social relationships that we have and those that we want” [1]. Due to the severe health effects of loneliness, estimated to be the same as smoking 15 cigarettes a day, the World Health Organization (WHO) has classified loneliness as a global health threat [2,3]. Although loneliness might affect people of any age, some age groups are more vulnerable. Older adults (aged 65 years or older) constitute a group that is more likely to experience life changes that may increase the risk of loneliness, such as the loss of a partner or close friends, as well as physical and psychological decline. Accordingly, loneliness is estimated to affect between 5% and 50% of the older population [4]. A large body of research has focused on elucidating risk factors of loneliness in old age [5]. However, while the individual risk factors of loneliness are well-researched [5], fewer studies have focused on the contextual factors [6]. These factors are not only important in themselves, but contextual factors are also likely to influence the individual factors. The digitalization of society is such a contextual factor.

Digital devices, such as computers, cell phones, and tablets, can increase access to social contact by allowing older adults to maintain relationships despite changing health and social circumstances [7-11]. Likewise, digital environments such as social media can offer opportunities to form new connections and relationships [8]. Digital environments can also strengthen the perceived availability of social support and connectedness to society [8]. If these digitally mediated interactions align with individuals’ relational goals and provide meaningful forms of relationships or support, the discrepancy between desired and actual social relationships may diminish and thereby decrease loneliness.

On the other hand, the digitalization of society might have negative psychological consequences [12]. It may exacerbate loneliness [13] by widening the gap between desired and actual social relationships. For those groups with limited digital skills, which are more common in old age, the shift to digital communication can reduce access to meaningful interactions [8,14], especially if digital communication replaces in-person communication [15]. In particular, older adults might find it difficult to use digital devices or feel limited by worries and fears about online safety risks [16]. In addition, digital environments may provide interactions that are frequent but lack the emotional depth or intimacy individuals desire [2,17,18], increasing the discrepancy between the quality of contact sought and the contact actually experienced. Digital environments also often contain idealized images of social connectedness, leading individuals to revise their expectations upward and perceive their own relationships as insufficient [19,20]. In summary, digitalization may intensify perceptions of unmet relational needs and hence lead to increased loneliness [12,13,21].

Recent reviews suggest that the use of digital devices, including social media use, is associated with lower levels of loneliness in old age, although not always strongly [21-24] and not consistently [15]. It has been shown that for specific groups, such as caregivers, the use of digital devices may directly reduce loneliness as well as buffer against the negative effects of loneliness [9]. Still, there is a lack of methodological robustness in the literature [22-24]. Few current studies have quantitative designs, and the studies with a quantitative design have small sample sizes and generally fail to control for established risk factors for loneliness [17,22-24]. Another limitation is that the majority of the studies are cross-sectional, which means that it is not possible to address questions about causality and change over time.

Some previous prospective and longitudinal studies have been identified. In a randomized controlled study, a computer intervention among chronically ill women showed no significant difference in the level of loneliness between those who received the intervention and the control group after the intervention [25]. On the other hand, a randomized field trial demonstrated that an internet-based intervention for older adults led to a significant reduction in perceived loneliness at 6 months [26]. However, this effect was not sustained at the 12-month follow-up. The evidence from population-based longitudinal studies is not clear either. In the Health and Retirement Study (HRS), which is based on a US sample, higher internet use was associated with decreased loneliness over an 8-year period [27], while in the longitudinal New Zealand Health, Work, and Retirement Study, a more diverse picture was observed [28]. Using the internet for social purposes was associated with decreased loneliness over a 4-year period, while informational and instrumental uses showed no significant relationship with loneliness over the same time period [28]. Access to and use of digital devices has also prospectively been linked to decreased risk of social isolation [29]. The diverse results might be a reflection of data assessed during different time periods and with different assessments.

In the years that modern cell phones (1990s), smartphones (2007), and tablets (2010) were introduced and then integrated into society, the timing of data collection played a vital role in any investigation of the relationship between use of digital devices and loneliness. Therefore, there is a need for more studies that explore the association between digital devices and changes in loneliness over time, particularly during the period that saw the introduction and adoption of smartphones and the rapid expansion of online communities in the mid-2000s, as these might serve as natural experiments (quasi-experimental design). If well-established risk factors for loneliness are also taken into account, such as age, gender, living circumstances (ie, living alone or not and/or civil status), education, and health, there is potential to explore the effect of use of digital devices on loneliness.

The longitudinal Swedish Adoption/Twin Study of Aging (SATSA) [30] provides such an opportunity. Hence, the aim of this study is to explore how daily use of digital devices is associated with change in loneliness over time, using data from a national longitudinal population-based study during a time when digital technology and digital environments were introduced and rapidly evolving, including measurement of established risk factors for loneliness.

## Methods

### Participants

Data came from SATSA [30], a longitudinal cohort study of aging, which is part of the Swedish Twin Registry [31,32]. Recruitment, eligibility, and testing procedures have been described in detail previously [30,33,34]. In brief, starting in 1984, twins who reached 50 years of age were recruited from the population-based Swedish Twin Registry for in-person testing and questionnaire completion [32]. The first questionnaire was collected in 1984 (Q1), including twins who had been reared apart and a matched sample of twins who had been reared together. The present study includes repeated longitudinal observations with a baseline in 2004 (Q5), when items concerning access and use of computers and cell phones were first included, and the subsequent surveys in 2007 (Q6), 2010 (Q7), 2012 (Q8), and 2014 (Q9). There were 794 participants in Q5, and 771 individuals (97.10%) had data for all of the variables of interest. Numbers for the subsequent waves were 648, 568, 463, and 414 (survey participants in waves Q6, Q7, Q8, and Q9) [34] and 645, 549, 458, and 411 (participants with data on all variables of interest in waves Q6, Q7, Q8, and Q9). Survey nonparticipation primarily resulted from illness or mortality. Mean age at baseline (Q5) was 69.64 (SD 11.00) years, and 60.05% of the sample was female. A subsample of individuals with 3 or more waves of participation was used for growth mixture models (N=492). The mean age at Q5 in the subsample was 66.55 (SD 9.60) years, and 61.58% of the subsample was female.

### Measures

The questionnaires were mailed to participants and completed using paper-and-pencil forms. No missing data were replaced using statistical estimation methods.

#### Digital Use Measures

Four questions about digital use were in the questionnaire waves: do you have access to a cell phone? If “yes,” how often do you use a cell phone? Do you have access to a computer? If “yes,” how often do you use a computer? Response options for the usage questions were daily, a few times a week, or more seldom. A daily digital use composite was created from the usage items. The value was 1 if the participant reported using either a cell phone or a computer on a daily basis and 0 if the participant did not use a digital device on a daily basis.

#### Loneliness

Following the harmonization procedures used by the Interplay of Genes and Environment across Multiple Studies (IGEMS) consortium [35], loneliness was assessed using 2 items. Participants were asked, “Are you ever troubled by feelings of loneliness?” and as part of the Center for Epidemiological Studies–Depression (CES-D) [36], participants were asked, “How often in the last week have you felt lonely?” Response options for both items ranged from 1 (almost never) to 4 (almost always). Items were summed to create a loneliness scale ranging from 2 to 8.

#### Demographic Measures

Education was categorized into 9 levels using the International Standard Classification for Education (ISCED) [37]. Thus, in the current sample, values ranged from 1 (primary education) to 6 (bachelor’s degree). To determine living situation, participants were asked about marital status and with whom they lived (eg, spouse, children, and others). If participants did not indicate that they were currently married and listed no other individuals living with them, they were categorized as living alone. The living situation was coded independently for Q5 through Q9.

#### Covariates

Self-rated health (SRH) was assessed with a single item: “How do you rate your general state of health?” Response options were good (3), fair (2), and bad (1). Depressive symptoms were measured using the CES-D scale [36]. Higher scores indicated more depressive symptoms. Openness to experience was measured as part of the neuroticism, extraversion, and openness personality inventory [38]. Six items measuring openness were included, with response options ranging from 1 (strongly disagree) to 5 (strongly agree). Scores could range from 6 to 30, and higher scores indicated more openness. Extraversion was assessed as part of the Eysenck Personality Questionnaire [39]. Nine yes/no items were summed to create an extraversion score; higher scores indicated greater extraversion. Perceived social support was a sum of 12 items asking about satisfaction with the number of social interactions (number of friends in your neighborhood, friends who drop by, friends you can turn to, family members you can turn to, etc) [40]. Responses were dichotomized: participants were either satisfied (1) or perceived that they had too little or too much social interaction (0). Scores ranged from 0 to 12, and 65.67% reported the maximum score of 12, indicating satisfaction with all social interactions.

### Statistical Method

The statistical analysis involved 4 steps. In the first step of the analysis, independent-samples *t* tests were used to compare loneliness in the full sample between individuals who did and did not use a digital device daily at each questionnaire wave. Because the twin nature of the data could reduce variability and thus inflate the likelihood of statistically significant differences, the significance level was set at *P*=.01. Second, growth mixture models (GMMs) were used to identify latent classes of longitudinal trajectories of loneliness in the subsample with 3 or more waves of participation. Using at least 3 waves of data in GMM is necessary for model identification and is recommended for obtaining reliable and unbiased estimates for latent subgroups [41]. GMM is a multilevel modeling technique similar to structural equation modeling (SEM) and hierarchical linear growth modeling that allows for the empirical identification of subgroups with homogeneous trajectories from a large, heterogeneous sample with longitudinal data [41]. The current analyses used time-based growth curve models to estimate the trajectories of change in loneliness from 2004 to 2014. For each class, the intercept and linear change over time were estimated, as well as the proportion of the sample in that class. Models were adjusted for sibling relatedness by modeling both between- and within-twin-pair variance in the random effects. Loss to follow-up was assumed to be missing at random (MAR); that is, not associated with any study variables, with the exception of age. Selection of the best model involved multiple factors, including model convergence, examining Akaike information criterion (AIC), Bayesian information criterion (BIC), sample size–adjusted BIC (SABIC), Lo-Mendell-Rubin adjusted likelihood ratio (LMR-LRT) tests of nested models [42], entropy, and substantive considerations [43]. However, fit statistics can be inconsistent [44], and in samples of 500 or more participants, BIC and SABIC are recommended [45]. GMMs were fitted using the heterogeneous linear mixed models (HLME) function in R (version 4.3.2, R Foundation for Statistical Computing) [46].

Third, analysis of variance and chi-square tests were used to examine differences between identified latent classes in demographic characteristics, daily digital use, and selected covariates. If the analysis of variance indicated significant mean differences between classes, post hoc Tukey tests were conducted to identify the differences. Finally, multinomial logistic regression was used to identify the variables that were associated with class membership, in the context of other related variables, with the largest class used as the reference class. The global null hypothesis that none of the predictors were significant was tested using the likelihood ratio chi-square test. The significance of individual predictors was tested using the Wald chi-square test, and odds ratios and their 95% CIs were examined. Linear regressions predicting the probability of class membership were also conducted. Class comparisons and regressions were conducted using SAS (version 9.4, SAS Institute) [47].

### Ethical Considerations

This study is a secondary analysis of previously collected data from the SATSA, a longitudinal research program designed to investigate genetic and environmental influences on aging. Ethical approval for the longitudinal data collection and analyses of SATSA data was obtained at several stages from the Research Ethics Committee at Karolinska Institutet and the Regional Ethics Review Board in Stockholm (Dnr 2007/151-31/4, 2010/657-31/3, and 2015/1729-31/5). The present analyses used previously collected SATSA data (2015/1729-31/5). The data provided to the authors were coded and did not contain names or personal identification numbers. The key linking study identification numbers to personal identification numbers was maintained separately and was not accessible to the authors. No attempt was made to identify individual participants, and all data were handled in accordance with applicable data protection requirements.

## Results

### Descriptive Statistics

Descriptive statistics for the full sample and the subsample with 3 or more waves of participation are presented in Table 1. The mean age in the subsample was 3 years younger than in the full sample, suggesting that those who were unable to complete 3 or more waves of participation were likely to be older. In the full sample, 86.25% (665/771) were aged 65 years or older during at least one wave of participation; the proportion was 87.60% (431/492) in the subsample. The lower age in the subsample may partly explain why the subsample had higher education, self-rated health, openness to experience, digital use, and lower values for depression, loneliness, and percentage living alone. However, these differences tend to be quite small in magnitude, with an average estimated effect size of 0.08. Figure 1 presents changes over time in ownership and use of cell phones and computers. Daily use of a cell phone increased from 16.62% (128/771) in 2004 to 45.81% (188/411) in 2014, and daily use of a computer increased from 18.85% (134/711) to 37.06% (152/411). Comparisons of early adopters (individuals reporting daily use of a cell phone or computer at Q5) vs later adopters (individuals without daily use of either a cell phone or computer at Q5) are reported in Table 2. Early adopters were significantly less likely to be female and less likely to live alone than later adopters. Early adopters were significantly younger and had higher SRH, fewer depressive symptoms, and greater openness and extroversion than later adopters, on average. Comparisons of mean loneliness for participants who did and did not report daily use of digital devices are presented in Figure 2. Independent-samples *t* tests confirmed that daily users of digital devices reported significantly lower levels of loneliness at each survey wave; full results of *t* tests are presented in Table S1 in Multimedia Appendix 1.

**Table 1:** Descriptive statistics for the full analytic sample and the subsample at baseline with ≥3 waves of participation across 5 questionnaire waves (Q5-Q9; 2004-2014) of the Swedish Adoption/Twin Study of Aging (SATSA).

Measures	Full sample (N=771)	3+ waves participation (n=492)
Daily digital use at Q5^a^, n (%)	203 (26.32)	162 (33.00)
Female, n (%)	463 (60.05)	303 (61.59)
Living alone at Q5, n (%)	257 (33.29)	134 (27.24)
Age range at Q5 (years)	45-96	45-92
Age at Q5 (years), mean (SD)	69.64 (11.00)	66.55 (9.60)
Waves participation, mean (SD)	3.37 (1.56)	4.39 (0.83)
ISCED^b^, mean (SD)	2.30 (1.64)	2.52 (1.73)
SRH^c^ at Q5, mean (SD)	2.51 (0.58)	2.59 (0.53)
CES-D^d^ at Q5, mean (SD)	35.49 (6.87)	34.99 (6.16)
Openness at Q5, mean (SD)	18.12 (4.22)	18.44 (3.90)
Extraversion at Q5, mean (SD)	5.19 (2.25)	5.23 (2.22)
Loneliness at Q5, mean (SD)	3.23 (1.32)	3.11 (1.22)
Social support at Q5, mean (SD)	10.89 (2.24)	10.79 (2.37)

^a^Q5: questionnaire wave 5.

^b^ISCED: International Standard Classification of Education.

^c^SRH: self-rated health.

^d^CES-D: Center for Epidemiological Studies Depression scale.

**Figure 1:**
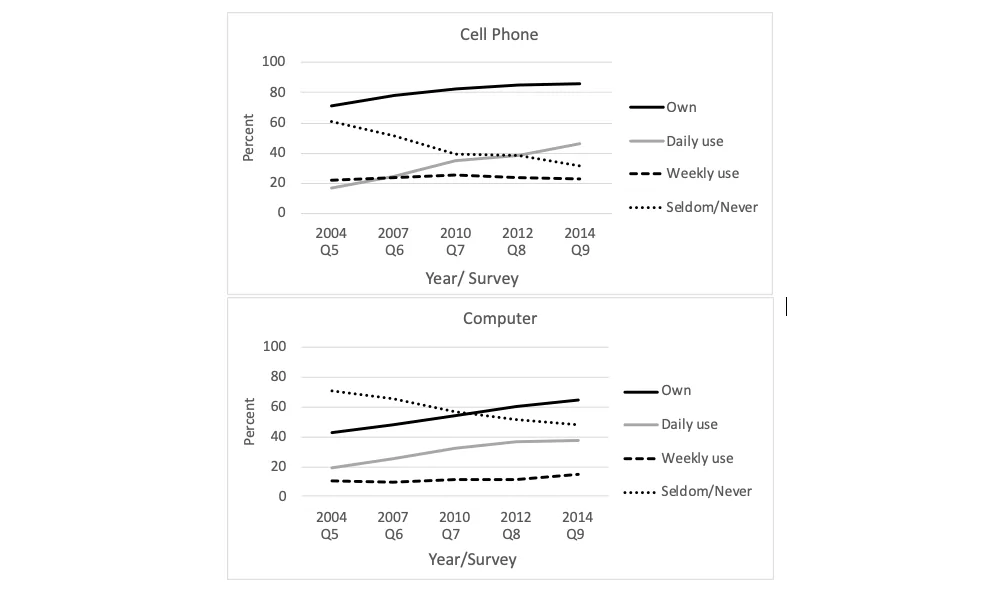
Ownership and usage of cell phones and computers from Q5 (2004) through Q9 (2014) in the full sample of the Swedish Adoption/Twin Study of Aging (SATSA). Sample sizes at each wave: Q5=771, Q6=645, Q7=549, Q8=458, and Q9=411.

**Table 2:** Comparison of characteristics of persons with daily use of digital devices vs nondaily use at baseline (Q5, 2004) in the Swedish Adoption/Twin Study of Aging (SATSA).

Variable	Less than daily digital use at Q5	Daily digital use at Q5	Test of group differences	*P* value
Participants, n	550	203	—^a^	—
Female (sex), n (%)	360 (65.51)	92 (45.37)	25.57 (1)^b^	<.001
Live alone, n (%)	211 (38.37)	49 (23.95)	11.01 (1)^b^	<.001
Age (years), mean (SD)	72.72 (10.00)	60.81 (8.34)	15.13 (751)^c^	<.001
ISCED^d^, mean (SD)	1.99 (1.43)	3.20 (1.86)	9.45 (751)^c^	<.001
SRH^e^, mean (SD)	2.45 (0.59)	2.71 (0.50)	5.65 (751)^c^	<.001
CES-D^f^, mean (SD)	35.97 (6.74)	34.55 (6.77)	2.57 (751)^c^	.01
Openness, mean (SD)	17.53 (4.25)	19.55 (3.73)	5.92 (751)^c^	<.001
Extraversion, mean (SD)	4.98 (2.25)	5.81 (2.13)	4.58 (751)^c^	<.001
Social support, mean (SD)	10.95 (2.17)	10.75 (2.47)	1.10 (751)^c^	.27
Loneliness, mean (SD)	3.39 (1.38)	2.83 (1.05)	5.24 (751)^c^	<.001

^a^Not applicable.

^b^Chi-square (*df*) value.

^c^*t* test (*df*) value.

^d^ISCED: International Standard Classification of Education.

^e^SRH: self-rated health.

^f^CES-D: Center for Epidemiological Studies Depression scale.

**Figure 2:**
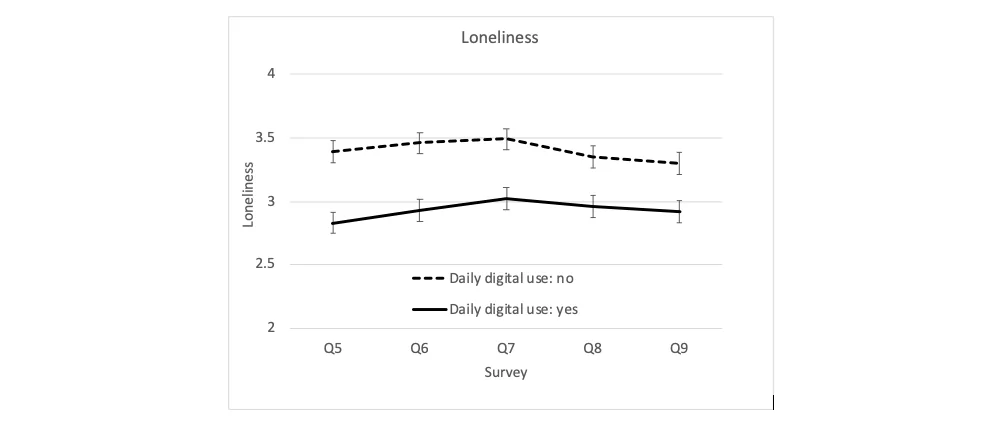
Comparison of mean loneliness for late middle–aged and old-aged individuals who did or did not report daily use of cell phones or computers across the 5 questionnaire waves of the Swedish Adoption/Twin Study of Aging (SATSA).

### Identifying Latent Classes

Results of fitting 4 GMMs to the longitudinal loneliness data are presented in Table 3, and parameter estimates from the 4 models are presented in Table S2 in Multimedia Appendix 1. The first model fitted the time-based linear growth curve model to the whole sample. The second model estimated 2 classes and fitted significantly better than the first model (Lo-Mendell-Rubin likelihood ratio test [LMR-LRT]=106.30, *df*=3; *P*<.001). Class 1 from this model, including 87.21% (429/492) of the sample, had a lower intercept and no significant change over time, and class 2 (63/492, 12.79%) had a higher intercept and a significant increase in loneliness over time. The third model estimated 3 classes and fit significantly better than the 2-class model (LMR-LRT=32.53, *df*=3; *P*<.001). The fourth model estimated 4 classes and did not improve model fit over the 3-class model. The SABIC model fit statistic was minimized for the 3-class model, and entropy was less than the generally accepted 0.60 cut-off [48] in the 4-class model; therefore, the 3-class model was selected as the best-fitting model. Longitudinal trajectories estimated for the 3 classes are presented in Figure 3; raw longitudinal trajectories are presented in Figure S1 in Multimedia Appendix 1. Class 1 (51/492, 10.37%) had the highest intercept and significant increases in loneliness over time. Class 2 (162/492, 32.93%) had an intermediate intercept and no significant change in loneliness over time. Class 3 (279/492, 56.71%) had the lowest intercept and also no significant change in loneliness over time.

**Table 3:** Model-fitting results for growth mixture modeling to identify latent classes of loneliness trajectories across 5 waves covering 10 years (2004-2014) in the Swedish Adoption/Twin Study of Aging (SATSA).

Model	Parameters, n	Log likelihood	LMR-LRT^a^	SABIC^b^	Entropy
1 class	6	–3373.23	—^c^	6764.98	1
2 classes	9	–3320.08	106.30^d^	6667.95	0.84
3 classes	12	–3302.94	32.53^d^	6642.93	0.77
4 classes	15	–3302.94	0.00	6652.20	0.53

^a^LMR-LRT: Lo-Mendell-Rubin likelihood ratio test comparison to previous model.

^b^SABIC: sample size–adjusted Bayesian information criterion.

^c^Not applicable.

^d^*P*<.01

**Figure 3:**
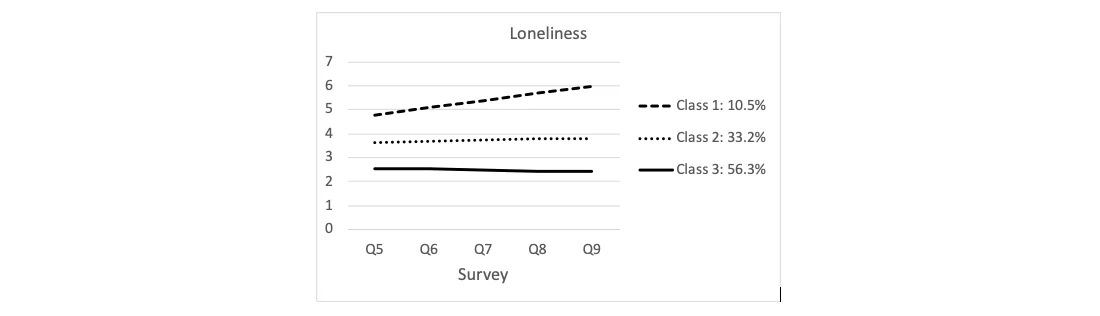
Estimated trajectories in the 3 latent classes of loneliness identified by growth mixture models, and percent of sample in each class.

### Comparing Latent Classes

The next step of the analyses was to determine how the 3 classes identified by GMM differed on demographic, personality, and daily digital use variables. Results presented in Table 4 show that the 3 classes differed significantly on every variable included, with the exception of openness to experience. Follow-up comparisons (Tukey post hoc comparisons of means) did not identify any particular class differences in mean participation rate across the study or mean ISCED. The 3 classes differed significantly from each other on age at Q5, CES-D at Q5, and daily digital use at Q5: the class with higher levels of loneliness had older participants, who reported more depressive symptoms and nondaily digital use. Class 1 and class 2 did not differ significantly on SRH, perceived social support, or extraversion, but class 3 had significantly higher SRH, perceived social support, and extraversion.

**Table 4:** Comparison of differences in covariates assessed at baseline in 2004 across the 3 latent classes of loneliness trajectories over 10 years. Class 1 (high increasing loneliness), class 2 (intermediate stable loneliness), and class 3 (low stable loneliness).

Variable	Class 1 high increasing (n=51)	Class 2 intermediate stable (n=162)	Class 3 low stable (n=279)	Statistical test of loneliness longitudinal class differences	*P* value
Daily digital use Q5^a^, n (%)	4 (7.84)	40 (24.69)	120 (43.01)	31.68 (2)^b^	<.001
Female, n (%)	40 (78.18)	111 (68.39)	150 (53.90)	17.10 (2)^b^	<.001
Waves participation, mean (SD)	4.11 (0.90)	4.25 (0.86)	4.41 (0.82)	3.84 (2, 489)^c^	.02
Live alone Q5, n (%)	29 (56.86)	60 (37.04)	45 (16.13)	47.81 (2)^b^	<.001
Live alone Q9^d^, n (%)	41 (79.41)	87 (53.72)	69 (24.89)	54.00 (2)^b^	<.001
Age at Q5, mean (SD)	73.25 (10.54)	68.02 (9.87)	64.48 (8.52)	22.64 (2, 489)^c^	<.001
Age range at Q5 (years)	45-92	46–89	45-86	—^e^	—
ISCED^f^, mean (SD)	2.11 (1.61)	2.40 (1.76)	2.67 (1.71)	3.08 (2, 489)^c^	.04
SRH^g^ at Q5, mean (SD)	2.43 (0.57)	2.49 (0.56)	2.68 (0.49)	9.40 (2, 489)^c^	<.001
CES-D^h^ at Q5, mean (SD)	40.18 (6.17)	36.72 (6.36)	33.03 (5.11)	45.72 (2, 489)^c^	<.001
Openness at Q5, mean (SD)	18.74 (3.77)	18.14 (4.11)	17.76 (3.80)	1.97 (2, 489)^c^	.14
Extraversion at Q5, mean (SD)	4.78 (1.95)	4.80 (2.31)	5.56 (2.16)	7.43 (2, 489)^c^	<.001
Social support at Q5, mean (SD)	9.56 (3.40)	10.30 (2.71)	11.28 (1.75)	16.86 (2, 489)^c^	<.001

^a^Q5: questionnaire wave 5.

^b^Chi-square (*df*) value.

^c^*F* test (*df*) value.

^d^Q9: questionnaire wave 9.

^e^Not applicable.

^f^ISCED: International Standard Classification of Education.

^g^SRH: self-rated health.

^h^CES-D: Center for Epidemiological Studies Depression scale.

Given the interrelationships among the demographic and daily digital use variables reported in Table 4, multinomial logistic regression was used to identify the variables that were associated with class membership in the context of the other variables. Variables included in the multinomial logistic regression were age, sex, ISCED, SRH, living situation, perceived social support, extraversion, and daily digital use, all derived from Q5. The analysis focused on baseline variables as predictors of future changes in loneliness, and particularly daily digital use. Results of multinomial logistic regression to predict membership in each class are reported in Table 5. The reference class was class 3, the largest class with low, stable loneliness over time. The likelihood ratio chi-square test of the global null hypothesis was significant (χ^2^_16_=137.26; *P*<.001). All variables except ISCED contributed to the prediction of class membership. Older adults were 9% more likely to belong to class 1 and 3% more likely to belong to class 2 compared with class 3. Females were 59% more likely to belong to class 2 than class 3. Individuals with higher SRH were 37% less likely to belong to class 2 versus class 3. Individuals who lived alone had much higher odds of belonging to class 1 and class 2 compared to class 3. Satisfaction with social support decreased the odds of belonging to class 1 by 28% and class 2 by 20% compared with class 3. Higher extraversion decreased the odds of belonging to class 2 compared with class 3 by 10%. Finally, individuals with daily digital use are 73% less likely to be in class 1. In a follow-up analysis comparing class 1 to class 2, the only variables that significantly predicted class membership were age and social support (see Table S3 in Multimedia Appendix 1).

**Table 5:** Multinomial logistic regression to predict membership in the 3 latent classes in longitudinal loneliness trajectories over 10 years, 2004-2014. Class 1 (high increasing loneliness), class 2 (intermediate stable loneliness), and class 3 (low stable loneliness) are reference categories.

Variable		Estimate (SE)			Wald *χ*^2^ (*df*=1)		Significance		Odds ratio (95% CI)
Age at Q5^a^									
	Class 1		0.08 (0.02)	15.97		<.001		1.09 (1.04-1.13)	
	Class 2		0.03 (0.01)	6.13		.01		1.03 (1.01-1.06)	
Sex (1=male, 2=female)									
	Class 1		0.53 (0.40)	1.72		.19		1.69 (0.77-3.71)	
	Class 2		0.46 (0.23)	3.87		.049		1.59 (1.00-2.51)	
ISCED^b^									
	Class 1		–0.12 (0.12)	1.05		.31		0.89 (0.71-1.11)	
	Class 2		–0.03 (0.07)	0.18		.67		0.97 (0.85-1.11)	
SRH^c^ at Q5									
	Class 1		–0.58 (0.32)	3.27		.07		0.56 (0.30-1.05)	
	Class 2		–0.47 (0.21)	4.93		.03		0.63 (0.42-0.95)	
Live alone at Q5									
	Class 1		1.46 (0.37)	15.78		<.001		4.30 (2.09-8.84)	
	Class 2		0.86 (0.25)	11.79		<.001		2.36 (1.45-3.86)	
Perceived social support at Q5									
	Class 1		–0.33 (0.07)	23.53		<.001		0.72 (0.62-0.82)	
	Class 2		–0.22 (0.05)	17.47		<.001		0.80 (0.72-0.89)	
Extraversion at Q5									
	Class 1		–0.06 (0.08)	0.43		.51		0.95 (0.80-1.12)	
	Class 2		–0.10 (0.05)	3.85		.0498		0.90 (0.81-1.00)	
Daily digital use at Q5 (0=no; 1=yes)									
	Class 1		–1.29 (0.60)	4.59		.03		0.27 (0.08-0.90)	
	Class 2		–0.38 (0.27)	1.91		.17		0.69 (0.40-1.17)	

^a^Q5: questionnaire wave 5.

^b^ISCED: International Standard Classification of Education.

^c^SRH: self-rated health.

As a sensitivity analysis, baseline loneliness was included in the model predicting class membership in trajectories of loneliness (see Table S4 in Multimedia Appendix 1). As expected, when using loneliness to predict membership in classes defined at least partly by loneliness at Q5, the contribution of most covariates was reduced. In this model, daily digital use was marginally significant (*P*=.06). Linear regressions predicting probability of class membership (reported in Table 5 in Multimedia Appendix 1) provided similar results. Older age, living alone, and less satisfaction with social support contributed significantly to the probability of being in class 1. Less satisfaction with social support contributed significantly to the probability of being in class 2. Lower age at Q5, better SRH, living with others, more satisfaction with social support, higher extraversion, and daily digital use contributed significantly to the probability of being in class 3.

## Discussion

### Principal Findings

This study contributes to the growing evidence [21-23] that daily use of digital devices is associated with lower levels of loneliness among late middle-aged and older adults. The unique contribution of this study is that we extend the findings from cross-sectional studies and show that there is a significant difference in the trajectories of loneliness over 10 years depending on whether late middle-aged and older adults used digital devices daily or less than daily. Daily use of digital devices was associated with decreased odds of belonging to the group with the lowest level of loneliness and also a stable level of loneliness, in comparison to those with high and increasing loneliness. In other words, those who did not use their digital devices daily were more likely to be in the group that had the highest levels of loneliness, which also increased over time. The influence of daily use of digital devices on the longitudinal trajectories of loneliness remained significant even when other well-established risk factors of loneliness were controlled for, such as age, gender, education, cohabitation, and self-rated health, but also for personality and perceived satisfaction with social support.

Three longitudinal latent classes of loneliness were identified: class 1 with high increasing loneliness (11%), class 2 with intermediate stable loneliness (33%), and class 3 with low stable loneliness (56%). As changes in loneliness are suggested to reflect individual experiences rather than a uniform age-related phenomenon [49], a latent class approach can capture distinct patterns of change that may be obscured when focusing solely on mean-level trajectories. An important finding was that the class with the highest levels of loneliness also appeared to be the most vulnerable to experiencing increasing loneliness over time. Thus, approximately one in ten late middle-aged and older adults with high levels of loneliness may benefit from interventions aimed not only at reducing their current levels of loneliness but also at preventing loneliness from worsening over time.

In line with previous prospective studies, which show that use of digital devices is associated with less change in loneliness over time [27,28], and that access to digital devices is associated with less perceived social isolation over time [29], daily digital use was associated with lower odds of being in the class with high and increasing levels of loneliness. The fact that daily use of digital devices is not only cross-sectionally associated with loneliness but also associated with change over time indicates that there might be a causal relationship in which use of digital devices decreases feelings of loneliness and may have a buffering effect on loneliness caused by health problems [9]. Although it is possible that the association operates in the opposite direction or that the relationship is bidirectional, it may also be confounded by other factors. We controlled for a broader range of potential confounders than many previous studies, including perceived social support, personality, and baseline loneliness. Importantly, the same pattern emerged in the sensitivity analyses, suggesting that the findings were robust to these adjustments.

While previous studies are performed in more recent contexts, our data are from a context when digitalization had not penetrated every aspect of society (2004-2014) in Sweden, and many persons did not have their own digital device. At baseline, about 40% had a computer and about 75% had a cell phone. In the first half of the 2000s, digital devices contributed mainly to social connectedness through being accessible remotely (compared with having a phone in the home) through text messages, emails, community sites, and chat forums. During the second half of the 2000s, what is commonly referred to as Web 2.0 began to gain real momentum. Smartphones were introduced (iPhone [Apple Inc] was introduced in the Swedish market in 2008), social media started to flourish (Facebook [Meta] was introduced in 2006 but reached the general public in 2009 and thereafter), and individuals also began to be connected to the internet on a daily basis. Although this limits the possibility of generalization to current cohorts, studying early adopters of digital technology provides a unique opportunity for a natural experiment, also commonly referred to as a quasi-experimental design. This provides stronger insight into causality, as when digital devices were first introduced, adoption was voluntary and unevenly distributed, likely creating larger contrasts between daily users and nonusers. In addition, as the digitalization had not penetrated all aspects of society, it also means that fewer interactions and communications were performed digitally, as fewer people had access to and used digital devices daily. In other words, people were less dependent on digital devices to remain in contact with family and friends. Finding an association in this context provides stronger evidence that daily use of digital devices is associated with lower levels of and less increase in loneliness over time.

Still, in the case of early adopters, it could be possible that the association between use of digital devices and loneliness is driven by personality. Specifically, extraversion and openness have been linked to general and specific use of digital devices in a meta-analysis [50]. Additionally, openness to new experiences has been associated with early adoption of digital devices [51] and use of digital devices in general [50], and extraversion has been associated with technology acceptance [52], in line with the current study. A meta-analysis supports that loneliness is related to both openness and extraversion [53]. In addition, a study on young adults (aged 18-25 years) shows an association between smartphone use and loneliness while controlling for personality [54], as in this study. However, levels of openness did not differ between the 3 identified classes of loneliness, but extraversion did. Still, the association between daily use of digital devices and loneliness is not driven by personality. To the best of our knowledge, we are the first to explore the influence of openness and extraversion on the association between digital use and loneliness in a sample that mainly contains older adults.

Potential explanations of why use of digital devices reduces loneliness include (1) the frequency and accessibility of social interactions increase, enabling individuals to maintain social relationships despite constraints related to geographical distance, limited mobility, or health conditions; (2) digital communication may also enhance perceived social support and a sense of societal connectedness; (3) digital environments facilitate the formation of new social ties, including both strong and weak connections, such as social media [10,21], but use of digital devices may also have a positive impact on self-esteem and self-worth [24,55]. When these digitally mediated interactions align with individuals’ relational needs and provide meaningful companionship or support, the discrepancy between desired and actual social relationships may be reduced, thereby potentially decreasing experiences of loneliness. Caution is warranted, as overuse of digital devices might have a negative effect. In a cross-sectional study among middle-aged and older Chinese adults, loneliness decreased when the number of hours of internet use increased, as long as it was not overuse (more than 5 hours per day) [56]. Internet addiction among older adults is a growing concern [57,58].

Various forms of social relationships have been associated with lower levels of loneliness [5], including cohabiting in the current study. Being married or being in a relationship is also usually linked to less loneliness. However, due to high collinearity between cohabiting (or not) and marital status, we did not explore marital status in itself. We are aware that some persons might be married but do not live together, for example, when one might be residing in a nursing home. However, we consider that these cases are not very likely to affect the results, as the majority of participants who cohabited lived with their partner. Additionally, we controlled for perceived satisfaction with social support, as social networks are an important factor for both loneliness and for adoption of digital technology in old age [16], where children and grandchildren might be important catalysts for use of digital devices [59], both as supporters to achieve and use digital devices, but also as maintaining contact with family and friends is an important reason for older adults to use digital devices [60]. The association between digital use and loneliness was not confounded by perceived satisfaction with social support, further strengthening the robustness of the association between digital use and loneliness.

Beyond daily use of digital devices and cohabitation, older age was associated with higher odds of belonging to the group characterized by higher and increasing levels of loneliness over a 10-year period. A review on risk factors of loneliness emphasized that the association between age and loneliness is common in bivariate analyses, but quite commonly age does not remain significantly associated with loneliness in multivariate analyses [5]. This is often explained by the association between higher age and higher loneliness co-occurring with other factors in old age, such as loss of their partner and declining health. This was not the case in the current study, despite the fact that we did control for various well-established risk factors of loneliness. It should be mentioned in the current study that the age range spans more than 50 years, larger than in most studies, which makes it more likely to find age differences. Regarding sex, the literature is inconclusive [5,61]. Therefore, the absence of an association with sex effect on loneliness in this study is not very surprising.

A recent cross-sectional Swedish study showed that lower self-reported mental health is strongly associated with higher levels of loneliness, even when other well-established risk factors of loneliness and use of digital devices are controlled for [62]. In this study, depressive symptoms were not related to class membership of loneliness. However, the assessments of mental health, here assessed by CES-D, do differ between the studies, which is a potential reason for the conflicting results.

### Strengths and Limitations

Strengths of this study are the rigorous longitudinal data collection with the use of an analytical method that incorporates multiple known risk factors of loneliness within the same model, allowing for a direct comparison of their impact on loneliness. Even if the longitudinal and quasi-experimental design provides stronger evidence for causality than many other designs, caution is still important.

An important note is that this study cannot be generalized to current contexts. However, showing an association between daily digital use and less loneliness and less increase in loneliness in a different context does strengthen the evidence. In contemporary studies, it is important to differentiate between the types of use in relation to loneliness, where it has been shown that use of digital devices for communication has a stronger negative correlation to loneliness [63] than use for information acquisition and entertainment [21]. However, as described above, at the time of the study, the digital environments were still limited, and people were less dependent on digital devices to remain in contact with family and friends, and few necessary services, like banking and contact with health care, had become digital and few, if any, had become “digital only.” It is more likely that the association between use of digital devices and loneliness reflects differences between early and late adopters of digital devices than differences between types of use, if it had been available. We have tried to rule out differences between early and late adopters by exploring a wide range of potential confounders, demographic, social, health-related, and personality, but with the same results. This supports the robustness of the association, although we cannot rule out that there might be other confounders that we have not controlled for.

To assess loneliness, being the primary outcome measure of this study, we followed the harmonization procedures used by the IGEMS consortium [35], taking all available data into account. As part of the harmonization process, the IGEMS loneliness measure was cross-validated with the University of California, Los Angeles (UCLA) loneliness scale in a crosswalk sample (N=888) [64]. Thus, despite including only 2 items, the loneliness scale used here has undergone rigorous validation testing. With a multi-item scale, it would have been possible to assess different aspects of loneliness, such as existential, emotional, and social loneliness. Although single loneliness items are by nature unidimensional, single items are highly correlated with one another and with multi-item scales [65]. Latent class analyses have the advantage of capturing distinct patterns of change that may be masked in studies focusing on mean-level trajectories. However, it is important to note that individual differences are not fully captured with a latent class approach, as 2 people may have similar levels and changes in loneliness while arriving at these experiences through very different circumstances [66].

Further, the generalizability is, as for the majority of studies on aging, impacted by selection bias for healthier older adults. Hence, the current results cannot be generalized to persons with more severe health conditions and persons living in nursing homes [67]. Furthermore, requiring at least 3 waves of data for inclusion in the latent class analysis, which is necessary for model identification, also introduced some selection bias. The differences between the full sample and the latent class sample were quite small on all variables of interest with the exception of age. The latent class sample was 3 years younger, on average, than the full sample. In other words, participants who were older at baseline were less likely to participate in 3 or more survey waves, with dropout resulting from illness or mortality. To the extent that changes in loneliness are associated with age, the latent class analysis may have underestimated age changes in loneliness. Thus, it is likely that the size of the latent class demonstrating increasing loneliness over time was underestimated in this sample. Although there is a selection bias, the attrition rate between the assessment waves in SATSA is fairly low, averaging 17% across waves, which strengthens the longitudinal findings.

Generalizability can also be discussed in relation to the fact that the participants are twins. However, empirical comparisons between twins and nontwins have shown that twins do not differ systematically from singletons on a wide range of psychological, behavioral, and health-related outcomes, nor in the associations between predictors and outcomes [68,69]. However, it can’t be ruled out that twins, and especially monozygotic twins, have a closer relationship to each other than nontwin siblings [70], which might contribute to fewer feelings of loneliness. However, in such a case, it is more likely to affect the level of loneliness than the associations. Additionally, the impact of being less lonely as a twin assumes that both twins in the couple are alive, which might not be the case in old age, and in the cases where one twin passes away, the surviving twin might experience greater loneliness.

### Conclusion

This study indicates that daily use of digital devices might be related to lower levels of loneliness and stable levels of loneliness, compared to those with the lowest use of digital devices, which showed higher levels and an increase in loneliness over time. The design suggests that digital devices might be a noninvasive tool to combat loneliness, although firm conclusions about the directionality cannot be drawn. As loneliness is a widespread health problem, using digitalization to tackle loneliness might have a substantial health benefit on the population level. Hence, it is important that older adults get support to overcome barriers, such as fear and worries, that commonly restrict older adults’ use of digital devices.

## Appendix

Multimedia Appendix 1 Supplementary figures and tables detailing device use loneliness trajectories, latent growth curve model parameters, class membership predictors, and longitudinal raw data.

## Abbreviations

AIC: Akaike information criterion
BIC: Bayesian information criterion
CES-D: Center for Epidemiological Studies–Depression
GMM: growth mixture model
HLME: heterogeneous linear mixed model
HRS: Health and Retirement study
IGEMS: Interplay of Genes and Environment across Multiple Studies
ISCED: International Standard Classification for Education
LMR-LRT: Lo-Mendell-Rubin likelihood ratio test
MAR: missing at random
SABIC: sample size–adjusted Bayesian information criterion
SATSA: Swedish Adoption/Twin Study of Aging
SEM: structural equation model
SRH: self-rated health
UCLA: University of California, Los Angeles
WHO: World Health Organization

## Data Availability

The datasets analyzed during this study are available in the National Archive of Computerized Data on Aging (NACDA) repository [71].
