# Supplementary material for: Early Adoption of Digital Devices and Change in Loneliness Over 10 Years Among Late Middle–Aged and Older Adults: Longitudinal Cohort Study
Source: JMIR Form Res. 2026 Sep 22;10:e95955. doi: 10.2196/95955 (PMC13643262; doi:10.2196/95955)
Supplement: Supplementary file 1 [file formative_v10i1e95955_app1.docx]

# Early Adoption of Digital Devices and Change in Loneliness Over 10 Years Among Late Middle-Aged and Older Adults: Longitudinal Cohort Study

Multimedia Appendix 1

Table S1. Mean loneliness in groups with and without daily use of cell phone or computer at each survey wave.

|  | Daily digital use: no | | Daily digital use: yes | |  |
| --- | --- | --- | --- | --- | --- |
| Survey | N | Mean (SD) | N | Mean (SD) | t-test |
| Q5 | 550 | 3.39 (1.38) | 203 | 2.83 (1.05) | 5.24 (df = 751)** |
| Q6 | 394 | 3.46 (1.46) | 220 | 2.93 (1.15) | 4.59 (df = 612)** |
| Q7 | 258 | 3.49 (1.37) | 241 | 3.02 (1.12) | 4.19 (df = 497)** |
| Q8 | 204 | 3.35 (1.41) | 229 | 2.96 (1.23) | 3.08 (df = 431)** |
| Q9 | 159 | 3.30 (1.28) | 221 | 2.92 (1.19) | 2.96 (df = 378)** |

** *p* < .01

Table S2. Latent growth curve parameter estimates (SE) for models with 1, 2, and 3 classes.

| Parameter | Intercept | Slope | Class n (%) |
| --- | --- | --- | --- |
| *Model 1* |  |  |  |
| Class 1 | 3.13 (0.06)** | 0.02 (0.01) | 492 (100.00) |
| *Model 2* |  |  |  |
| Class 1 | 3.01 (0.07)** | -0.04 (0.02)* | 429 (87.21) |
| Class 2 | 3.77 (0.20)** | 0.39 (0.05)** | 63 (12.79) |
| *Model 3* |  |  |  |
| Class 1 | 4.49 (0.25)** | 0.30 (0.07)** | 51 (10.50) |
| Class 2 | 3.59 (0.12)** | 0.04 (0.03) | 162 (33.20) |
| Class 3 | 2.59 (0.09)** | -0.04 (0.02) | 279 (56.30) |

* parameter is significantly different from zero at *p* < .05

** parameter is significantly different from zero at *p* < .01

Table S3. Logistic regression to predict membership in the latent class 2 vs. class 1 (reference).

| Variable | Estimate (SE) | Wald chi-square (df=1) | Significance | Odds Ratio | 95% C.I. |
| --- | --- | --- | --- | --- | --- |
| Age at Q5 |  |  |  |  |  |
| Class 2 | -0.04 (0.02) | 5.51 | 0.02 | 0.96 | 0.92, 0.99 |
| Sex (1=male, 2=female) | |  |  |  |  |
| Class 2 | -0.11 (0.41) | 0.08 | 0.78 | 0.89 | 0.41, 1.98 |
| ISCED |  |  |  |  |  |
| Class 2 | 0.05 (0.11) | 0.22 | 0.64 | 1.05 | 0.84, 1.32 |
| SRH at Q5 |  |  |  |  |  |
| Class 2 | 0.04 (0.31) | 0.02 | 0.89 | 1.04 | 0.57, 1.92 |
| Live alone at Q5 |  |  |  |  |  |
| Class 2 | -0.50 (0.35) | 2.02 | 0.16 | 0.61 | 0.30, 1.21 |
| Social Support at Q5 | | |  |  |  |
| Class 2 | 0.12 (0.06) | 4.15 | 0.04 | 1.12 | 1.00, 1.26 |
| Extraversion at Q5 | | |  |  |  |
| Class 2 | -0.05 (0.08) | 0.39 | 0.53 | 0.95 | 0.80, 1.12 |
| Daily digital use at Q5 (0=no, 1=yes) | | | | | |
| Class 2 | 0.98 (0.62) | 2.46 | 0.12 | 2.65 | 0.78, 8.98 |

Table S4. Multinomial logistic regression to predict membership in the 3 latent classes with baseline loneliness included. Reference = class 3 (low stable loneliness).

| Variable | Estimate (SE) | Wald chi-square (df=1) | Significance | Odds Ratio | 95% C.I. |
| --- | --- | --- | --- | --- | --- |
| Age at Q5 |  |  |  |  |  |
| Class 1 | 0.07 (0.02) | 9.84 | 0.00 | 1.08 | 1.03, 1.12 |
| Class 2 | 0.03 (0.01) | 3.78 | 0.05 | 1.03 | 1.00, 1.06 |
| Sex (1=male, 2=female) | |  |  |  |  |
| Class 1 | 0.66 (0.44) | 2.25 | 0.13 | 1.93 | 0.82, 4.56 |
| Class 2 | 0.48 (0.26) | 3.25 | 0.07 | 1.61 | 0.96, 2.71 |
| ISCED |  |  |  |  |  |
| Class 1 | -0.12 (0.13) | 0.91 | 0.34 | 0.89 | 0.69, 1.13 |
| Class 2 | -0.06 (0.08) | 0.56 | 0.45 | 0.94 | 0.81, 1.10 |
| SRH at Q5 |  |  |  |  |  |
| Class 1 | -0.54 (0.36) | 2.17 | 0.14 | 0.58 | 0.29, 1.19 |
| Class 2 | -0.45 (0.24) | 3.44 | 0.06 | 0.64 | 0.40, 1.03 |
| Live alone at Q5 | | |  |  |  |
| Class 1 | 0.31 (0.43) | 0.53 | 0.47 | 1.37 | 0.59, 3.17 |
| Class 2 | 0.15 (0.29) | 0.27 | 0.60 | 1.17 | 0.66, 2.07 |
| Perceive Social Support at Q5 | | | | | |
| Class 1 | -0.14 (0.08) | 3.03 | 0.08 | 0.87 | 0.74, 1.02 |
| Class 2 | -0.10 (0.06) | 2.52 | 0.11 | 0.91 | 080, 1.02 |
| Extraversion at Q5 | | |  |  |  |
| Class 1 | 0.05 (0.09) | 0.28 | 0.60 | 1.05 | 0.87, 1.26 |
| Class 2 | -0.02 (0.06) | 0.13 | 0.72 | 0.98 | 0.87, 1.10 |
| Loneliness at Q5 | | |  |  |  |
| Class 1 | 1.67 (0.21) | 65.58 | 0.00 | 5.33 | 3.55, 7.99 |
| Class 2 | 1.19 (0.15) | 66.31 | 0.00 | 3.30 | 2.48, 4.39 |
| Daily digital use at Q5 (0=no, 1=yes) | | | | | |
| Class 1 | -1.20 (0.64) | 3.54 | 0.06 | 0.30 | 0.09, 1.05 |
| Class 2 | -0.35 (0.31) | 1.30 | 0.25 | 0.70 | 0.38, 1.29 |

Table S5. Linear regressions predicting probability of class membership.

| Variable | Class 1  Parameter (SE) | Class 2  Parameter (SE) | Class 3  Parameter (SE) |
| --- | --- | --- | --- |
| Age at Q5 | 0.01 (0.00)** | 0.00 (0.00) | -0.01 (0.00)** |
| Sex | 0.03 (0.03) | 0.04 (0.04) | -0.07 (0.04) |
| ISCED | -0.01 (0.01) | 0.00 (0.01) | 0.01 (0.01) |
| SRH at Q5 | -0.03 (0.02) | -0.06 (0.03) | 0.09 (0.03)** |
| Live alone at Q5 | 0.11 (0.03)** | 0.08 (0.04) | -0.18 (0.04)** |
| Social Support at Q5 | -0.02 (0.00)** | -0.02 (0.01)** | 0.04 (0.01)** |
| Extraversion at Q5 | 0.00 (0.01) | -0.02 (0.01) | 0.02 (0.01)* |
| Daily digital Use at Q5 | -0.05 (0.03) | -0.04 (0.04) | 0.09 (0.04)* |
|  |  |  |  |
| Model Fit | F(8,473)=11.86** | F(8,473)=5.87** | F(8,473)=21.20** |
| Adjusted R^2^ | 0.15 | 0.08 | 0.25 |

* *p* < .05

** *p* < .01

Figure S1. Raw data for longitudinal changes in loneliness in the 3 latent classes.
